# miR-222 Is Involved in the Amelioration Effect of Genistein on Dexamethasone-Induced Skeletal Muscle Atrophy

**DOI:** 10.3390/nu14091861

**Published:** 2022-04-29

**Authors:** Mailin Gan, Jianfeng Ma, Jingyun Chen, Lei Chen, Shunhua Zhang, Ye Zhao, Lili Niu, Xuewei Li, Li Zhu, Linyuan Shen

**Affiliations:** 1 Department of Animal Science, College of Animal Science and Technology, Sichuan Agricultural University, Chengdu 611130, China; ganmailin@stu.sicau.edu.cn (M.G.); 2020202051@stu.sicau.edu.cn (J.M.); 2020302110@stu.sicau.edu.cn (J.C.); chenlei815918@sicau.edu.cn (L.C.); 14081@sicau.edu.cn (S.Z.); zhye@sicau.edu.cn (Y.Z.); niulili@sicau.edu.cn (L.N.); xuewei.li@sicau.edu.cn (X.L.); zhuli@sicau.edu.cn (L.Z.); 2 Farm Animal Genetic Resource Exploration and Innovation Key Laboratory of Sichuan Province, Sichuan Agricultural University, Chengdu 611130, China

**Keywords:** genistein, muscle atrophy, miR-222, *IGF1*

## Abstract

Skeletal muscle atrophy is a complex degenerative disease characterized by decreased skeletal muscle mass, skeletal muscle strength, and function. MicroRNAs (miRNAs) are a potential therapeutic target, and natural products that regulate miRNA expression may be a safe and effective treatment strategy for muscle atrophy. Previous studies have shown beneficial effects of genistein treatment on muscle mass and muscle atrophy, but the mechanism is not fully understood. Differential co-expression network analysis revealed that miR-222 was upregulated in multiple skeletal muscle atrophy models. Subsequent in vitro (C2C12 myoblasts) and in vivo (C57BL/6 mice) experiments showed that genistein could alleviate dexamethasone-induced muscle atrophy and downregulate the expression of miR-222 in muscle tissue and C2C12 myotubes. The dual-luciferase reporter assay system confirmed that *IGF1* is a target gene of miR-222 and is regulated by genistein. In C2C12 myotubes, both dexamethasone and miR-222 overexpression promoted muscle atrophy, however, this function was significantly reduced after genistein treatment. Furthermore, we also observed that both genistein and miR-222 antagomiR could significantly inhibit dexamethasone-induced muscle atrophy in vivo. These results suggest that miR-222 may be involved in the regulation of genistein on muscle atrophy, and genistein and miR-222 may be used to improve muscle health.

## 1. Introduction

Skeletal muscle mass accounts for approximately 40% of a healthy adult body weight and plays an important role in supporting body weight, maintaining posture, and maintaining body temperature [1]. In addition, it also acts as a secretory organ to secrete a variety of myokines to affect the function of other organs [2]. As the basis of skeletal muscle function, skeletal muscle mass is often affected by various factors such as exercise and disease [3]. Resistance exercise can increase protein synthesis in skeletal muscle cells and induce muscle hypertrophy; while limb disuse, heart failure, cachexia, Duchenne muscular dystrophy, etc. can lead to decreased skeletal muscle protein synthesis or enhanced degradation, causing skeletal muscle atrophy [4]. Impaired muscle function due to muscle atrophy is one of the important causes of increased mortality from wasting disease [5]. Therefore, the maintenance of muscle mass and function is of great significance to the health of the body.

The maintenance of skeletal muscle mass depends on the balance between skeletal muscle protein synthesis and breakdown [6]. Skeletal muscle hypertrophy occurs when skeletal muscle cell protein synthesis exceeds degradation, increasing the size of preexisting muscle fibers within skeletal muscle. Studies have shown that a variety of key molecules and signal transduction pathways regulate skeletal muscle hypertrophy, such as insulin-like growth factor 1 (*IGF1*)/phosphatidylinositol 3-kinase (*PI3K*)/*Akt*, Myostatin (*MSTN*), etc. [7,8]. When protein degradation exceeds protein synthesis, skeletal muscle atrophy occurs, characterized by decreased muscle fiber cross-sectional area and decreased muscle strength. There are two main protein degradation pathways in cells: the ubiquitin proteasome system (UPS) pathway and the autophagy-lysosome pathway [9]. The UPS pathway is the main pathway for the selective degradation of ATP-dependent proteins in cells, degrading 80% to 90% of ubiquitinated proteins in cells [10]. Numerous studies have shown that *MAFbx* (muscle atrophy F-box, also known as atrogin-1, *Fbxo32* (F-box protein 32)) and *MuRF1* (muscle RING finer 1, also known as *Trim63* (tripartite motif-containing 63)) are skeletal muscle-specific ubiquitin ligases that regulate protein ubiquitination and are used as important markers of various muscle atrophy [11].

There are various hormones in the body that act on the protein metabolism of skeletal muscle [12]. The hormones that promote muscle protein anabolism in the body mainly include growth hormone (*GH*), testosterone, estrogen, and *IGF1*, while the main ones that promotes muscle protein catabolism is glucocorticoids (GCs) [13]. In the treatment of muscle atrophy, in addition to physical therapy programs based on exercise, gene therapy, stem cell therapy and drug therapy programs are the main research directions for the treatment of muscular dystrophy. However, there is no effective pharmacological treatment for muscle atrophy, and gene therapy and stem cell therapy are still immature [14]. Studies have shown that estrogen is a potential drug for treating skeletal muscle atrophy [15]. Genistein is one of the phytoestrogens widely distributed in leguminous plants, which can show weak estrogenic or antiestrogenic activity in mammals. A previous study found that dietary genistein prevents denervation-induced muscle atrophy in male rodents by affecting estrogen receptor-α(*ERα*, also known as *ESR1*) [16], but the underlying mechanisms and regulatory networks remain unclear.

MicroRNAs (miRNAs) are a class of short noncoding RNAs (ncRNAs) consisting of about 22 nucleotides. As a class of epigenetic regulators, a variety of miRNAs have been identified to play critical roles in myogenesis, development, injury repair, and muscle atrophy [17]. Previous studies have found that genistein can regulate miRNAs involved in the treatment of cancer [18], muscle fiber type switching [19], NASH [20] and obesity [21], but it is unclear whether or which miRNAs are involved in the regulation of genistein on skeletal muscle atrophy.

In this study, we focused on the effect of genistein on dexamethasone-induced skeletal muscle atrophy and explored the potential mechanism of genistein-regulated miRNAs in the process of muscle atrophy. The results may provide new insights for genistein and miRNA in the prevention and treatment of muscle atrophy.

## 2. Materials and Methods

### 2.1. Animals and Treatment

50 ten-week female C57BL/6 mice (Chengdu Dashuo Experimental Animal Co, Ltd., Chengdu, China) were maintained in a dedicated animal room at 22 °C ± 3 °C under a natural light cycle. During the experiment, all mice were reared by specially-assigned persons, provided with sufficient feed and water. Animal feeding procedures and experimental protocols were carried out under the supervision of the Animal Care and Ethics Committee of Sichuan Agricultural University (No. 20210156, Chengdu, Sichuan, China).

### 2.2. Muscle Atrophy Model

**Figure 1:**
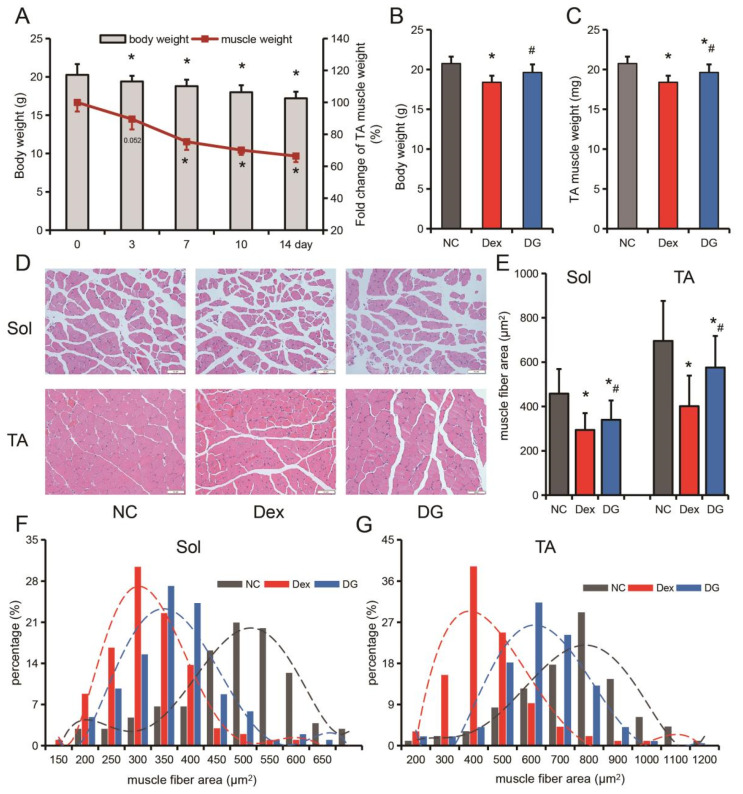
Genistein attenuates Dex-induced muscle fiber area reduction in mice. (**A**) Time course of mouse body weight and tibialis anterior (TA) muscle mass loss in a Dex-induced muscle atrophy model. (**B**) Mice body weight at the end of treatment. (**C**) TA muscle weight of mice at the end of treatment. (**D**) H-E (hematoxylin-eosin) staining of TA and Sol (soleus) muscle. (**E**) Mean muscle fiber area of TA and Sol muscle. (**F**,**G**) The distribution frequency of TA (**F**) and Sol (**G**) muscle fibers. A (pre-experiment, without genistein treatment), *n* = 3; (**B**–**G**) (formal experimental, NC: normal control group, Dex: Dexamethasone-treated group, DG: Dexamethasone+genistein treatment group), n = 6. * *p* < 0.05, compared with the NC group, # *p* < 0.05, compared with Dex group.

The animals were intraperitoneally injected with Dex (dexamethasone, 25 mg/kg body weight) after seven days of acclimatization to establish a muscle atrophy model [22]. Genistein (Purity ≥ 98%, Jingzhu Biotechnology, Nanjing, China) was taken orally at a dose of 10 mg/kg body weight per day, started 3 days before Dex treatment, and continued until the end of the experiment [19]. The miR-222 antagomir and negative control antagomir (GenePharma, Shanghai, China) were used. Intramuscular injection (25 nmol/mouse), once every 2 days for 10 days. Sample collection was performed the day after the injection procedure was completed. Dex (0.2 mL per mouse) was injected intraperitoneally every day at 6 pm and weighed before injection. In the preliminary experiment, 3 mice were euthanized on the 0th day, the 3rd day, the 7th day, the 10th day and the 14th day, and the tibialis anterior (TA) muscle was taken and weighed to evaluate the effect of muscle atrophy (Figure 1A). The control group was injected with an equal amount of phosphate-buffered saline (PBS). According to the pre-experimental results, the skeletal muscle atrophy model was constructed by intraperitoneal injection of Dex for 10 consecutive days in the formal experimental.

### 2.3. Cell Culture and Transfection

The C2C12 myoblast cell line and the HeLa cell line (Stem Cell Bank, Chinese Academy of Sciences, Beijing, China) were cultured using a carbon dioxide incubator at 37 °C in a 5% carbon dioxide system. C2C12 myoblasts were cultured in growth medium (GM, 10% FBS (fetal bovine serum, Gibco, Carlsbad, CA, USA) +90% DMEM (Gibco)) before differentiation, and changed to differentiation medium (DM, DMEM with 2% horse serum (Gibco)) when the cell density reached about 80%. The medium was changed every 24 h and transfected after 3 days in DM.

The genistein stock solution (1M) was dissolved in dimethyl sulfoxide (DMSO) and stored at room temperature at −20 °C [19]. To induce muscle atrophy in vitro, cells were incubated in DM added with 5 μM Dex for 48 h before harvesting or for morphological analysis [23]. At the same time, genistein (10 μM), miR-222 mimic (50 nM), inhibitor (50 nM) and negative control (50 nM, Ribobio, Guangzhou, China) were transfected into the C2C12 myotube using Lipofectamine 3000 (Invitrogen, Guangzhou, China). Transfection efficiency was detected using RT-qPCR (Real-Time Quantitative PCR).

### 2.4. Skeletal Muscle Tissue Section and C2C12 Myotube Immunofluorescence Staining

Skeletal muscle samples were freshly isolated and mounted in muscle fixation fluid (Servicebio, Wuhan, China). After the muscle tissues were fixed, the tissues were dehydrated with different concentrations of ethanol, embedded in paraffin, then sliced, and finally stained with H-E (hematoxylin-eosin), observed under a microscope and photographed. C2C12 myotubes were fixed with paraformaldehyde, blocked with goat serum for 1 h, and then incubated with *MYHC* primary antibody (Servicebio) at 4 °C for 12 h. Finally, the cells were incubated with Fitc-labeled secondary antibody for 2 h and observed under a fluorescence microscope (TE2000, Nikon, Tokyo, Japan). Measure the diameter of each myotube at the midpoint. Measurements of skeletal muscle fiber area and C2C12 myotube diameter were determined using the Count/Size tool in Image-Pro Plus 6.0 software (Media Cybernetics Corp., Bethesda, MD, USA).

### 2.5. Total RNA Extraction and RT-qPCR

Accurately weigh 30 mg of muscle tissue, add 1 mL of Trizol (TaKaRa, Dalian, China) to fully lyse, centrifuge at 3000 rpm for 5 min, and take the supernatant to extract total RNA according to the kit instructions. For C2C12 myotubes, after removing the medium, add 1ml Trizol (12-well cell culture plate) to the myotubes for sufficient lysis, and then extract total RNA according to the instructions. Total RNA was first reverse transcribed into cDNA and RT-qPCR was performed using the SYBR Premix Ex Taq kit (TaKaRa). U6 was used as a miRNA internal control, and *ACTB* served as an mRNA internal control. The primer sequences are listed in Table S1.

### 2.6. Dual Luciferase Reporting System

The psiCHECK™-2 vector inserts the 3’ UTR of IGF1, which is predicted to bind to miR-222. The vector, miR-222 mimic and negative control were transfected into HeLa cell lines using Lipofectamine 3000 (Invitrogen). 48 h after transfection, cells were collected and lysed, and luciferase activity was measured using a dual-luciferase detection system (Promega, Madison, WI, USA) according to the kit instructions.

### 2.7. Data Analysis in Public Databases

The miRNAs sequencing data used in this study were all derived from the GEO database. (https://www.ncbi.nlm.nih.gov/geo/, accessed on 1 March 2022). Target genes of miRNAs were predicted using TargetScan [24], RNAhybrid [25] and miRDB [26] programs. Gene interaction network analysis was performed using STRING 11.5 (https://cn.string-db.org/, accessed on 12 March 2022) and plotted using Cytoscape 3.8. Gene Ontology (GO) enrichment analysis was plotted by a free online platform for data analysis and visualization (http://www.bioinformatics.com.cn, accessed on 12 March 2022).

### 2.8. Statistical Analysis

Statistical analysis was performed using SPSS software (SPSS 20.0, SPSS Inc., Chicago, IL, USA) and expressed as means ± standard deviation (SD). One-way ANOVA was used to calculate the P value between the two groups, and *p* ≤ 0.05 was considered to be significantly different.

## 3. Results

### 3.1. Genistein Alleviates Dexamethasone-Induced Skeletal Muscle Atrophy In Vivo

In the pre-experiment, after dexamethasone (Dex) treatment, mice body weight and tibialis anterior (TA) muscle mass continued to decline, reaching significant levels on day 3 (19.40 g) and day 7 (75.37%), respectively (Figure 1A). Taking into account the treatment time and effect of Dex, the muscle atrophy model was constructed by continuous injection of Dex for 10 days in the subsequent experiments. Administration of genistein significantly inhibited Dex-induced loss of body weight and TA muscle mass (Figure 1B,C). We further measured the muscle fiber area of the soleus (Sol) muscle and TA muscle, and found that Dex treatment led to a decrease in the muscle fiber area of Sol and TA, while genistein significantly inhibited the decrease of muscle fiber area of Sol and TA caused by Dex treatment (Figure 1D–G). These results suggest that Dex-induced skeletal muscle mass reduction is mainly characterized by muscle fiber area reduction, and genistein improves muscle atrophy by inhibiting muscle fiber area reduction.

### 3.2. Genistein Alleviates Dexamethasone-Induced Skeletal Muscle Atrophy In Vitro

**Figure 2:**
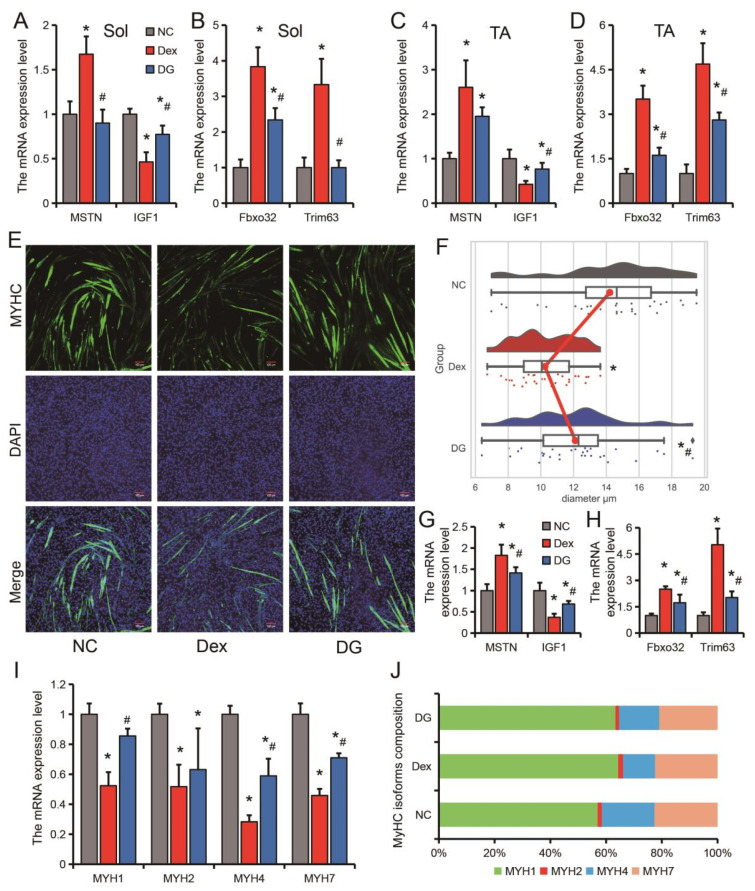
Genistein alleviates Dex-induced skeletal muscle atrophy in vitro. (**A**) The expression of *MSTN* and *IGF1* in Sol. (**B**) The expression of *Fbxo32* and *Trim63* in Sol. (**C**) The expression of *MSTN* and *IGF1* in TA. (**D**) The expression of *Fbxo32* and *Trim63* in TA. (**E**) Immunofluorescent staining for C2C12 myotubes. (**F**) Rain-cloud diagram showing C2C12 myotube diameter distribution. (**G**) The expression of *MSTN* and *IGF1* in C2C12 myotubes. (**H**) The expression of *Fbxo32* and *Trim63* in C2C12 myotubes. (**I**,**J**) The relative expression (**I**) and proportion (**J**) of each isoform of MYHC (myosin heavy chain). *n* = 6. * *p* < 0.05, compared with the NC group, # *p* < 0.05.

The expression levels of *MSTN*, *Fbxo32* and *Trim63* were all significantly increased, and the expression level of *IGF1* was significantly decreased in the Sol and TA muscles of Dex-treated mice (Figure 2A–D). Compared with the Dex group, genistein significantly inhibited the Dex-induced increase in the expression levels of *MSTN*, *Fbxo32* and *Trim63*, and the decrease in the expression level of *IGF1* (Figure 2A–D). To further clarify the regulatory effect of genistein on muscle fibers in muscle atrophy, we constructed an in vitro muscle atrophy model using differentiated C2C12 myoblasts. Similar to in vivo muscle atrophy model, Dex treatment resulted in a significant decrease in myotube diameter in the cellular model, while genistein attenuated the effect of Dex on C2C12 cells (Figure 2E,F). In the in vitro model, genistein also significantly inhibited the Dex-induced increases in the expression levels of *MSTN*, *Fbxo32* and *Trim63*, and promoted the expression of *IGF1* (Figure 2G,H). Further analysis of muscle fiber types in the cell model showed that all types of muscle fibers were significantly reduced after Dex treatment, and genistein significantly alleviated the reduction in the expression levels of *MYH1*, *MYH4* and *MYH7* (Figure 2I). Analysis of the *MYHC* isoforms composition revealed that Dex treatment mainly resulted in a decrease in the content of *MYH4*, which is a marker of fast twitch muscle (Figure 2J). Taken together, these data demonstrate that genistein is effective in both in vitro and in vivo muscle atrophy, suggesting the potential of genistein in preventing muscle atrophy.

### 3.3. miR-222 Is One of the Core Regulators of Muscle Atrophy

**Figure 3:**
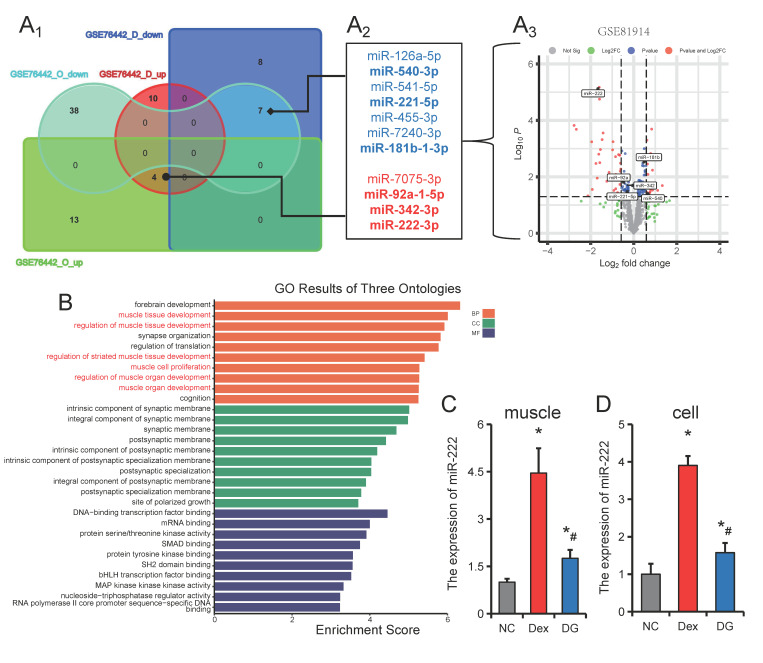
miR-222 is upregulated in multiple models of skeletal muscle atrophy. (**A_1_**–**A_3_**) Co-expression analysis of miRNAs in disuse atrophy models (GSE76442_D), old mice (GSE76442_D), and denervated muscle atrophy models (GSE81914). Data sourced from GEO DataSets (GSE76442, GSE81914). (**B**) Gene ontology analysis of miR-222 target genes. (**C**,**D**) The expression of miR-222 in skeletal muscle (**C**) and C2C12 myotubes (**D**). (**C**,**D**), *n* = 6. * *p* < 0.05, compared with the NC group, # *p* < 0.05.

Co-expression analysis of differentially expressed miRNAs in aging and disuse muscle atrophy revealed miR-126a-5p, miR-540-3p, miR-541-5p, miR-221-5p, miR-455-3p, miR-7240-3p and miR-181b-1-3p were downregulated in two muscle atrophy models, while miR-7075-3p, miR-92a-1-5p, miR-342-3p and miR-222-3p were upregulated in both muscle atrophy models. (Figure 3A_1_,A_2_). Further combined analysis with denervated muscle atrophy models found that miR-540-3p, miR-221-5p and miR-181b-1-3p were also down-regulated in denervated muscle atrophy models, while miR-92a-1-5p, miR- 342-3p and miR-222-3p are upregulated in a denervated muscle atrophy model (Figure 3A_3_). Among them, miR-222-3p had the smallest *p*-value and the largest differential change (Figure 3A_3_). To further explore the possible regulatory mechanism of miR-222 in muscle atrophy, we used TargetScan and miRBD to predict the target genes of miR-222. GO (Gene Ontology) analysis showed that miR-222 target genes were mainly involved in biological processes (BP) such as muscle tissue development, regulation of muscle tissue development, regulation of striated muscle tissue development, muscle organ development, regulation of muscle organ development and muscle cell proliferation (Figure 3B). Furthermore, we also found that genistein could inhibit Dex-induced upregulation of miR-222 both in vitro and in vivo (Figure 3C,D). These results suggest that miR-222 is a core regulator of muscle atrophy and is regulated by genistein (Table S2).

### 3.4. IGF1 Is an Important Target Gene of miR-222 and Is Regulated by Genistein

**Figure 4:**
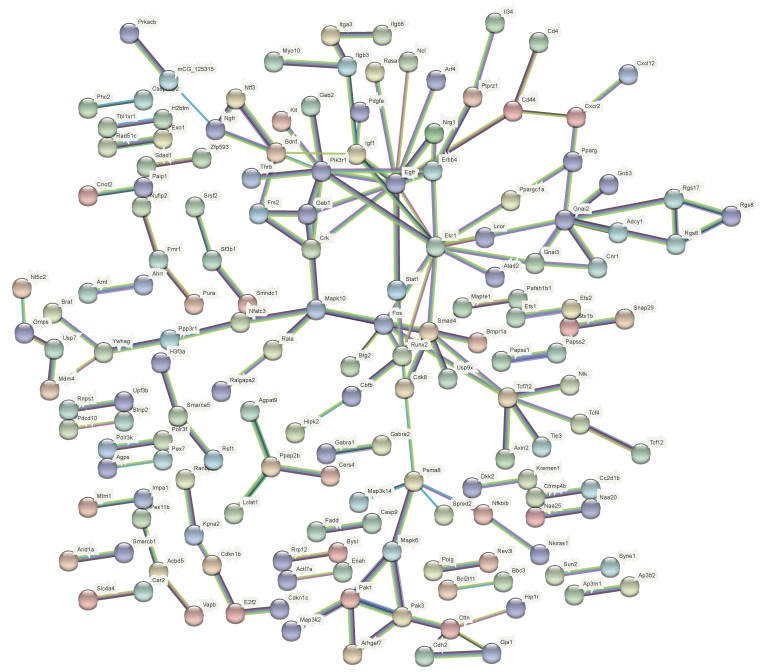
Interaction network of miR-222 target genes.

**Figure 5:**
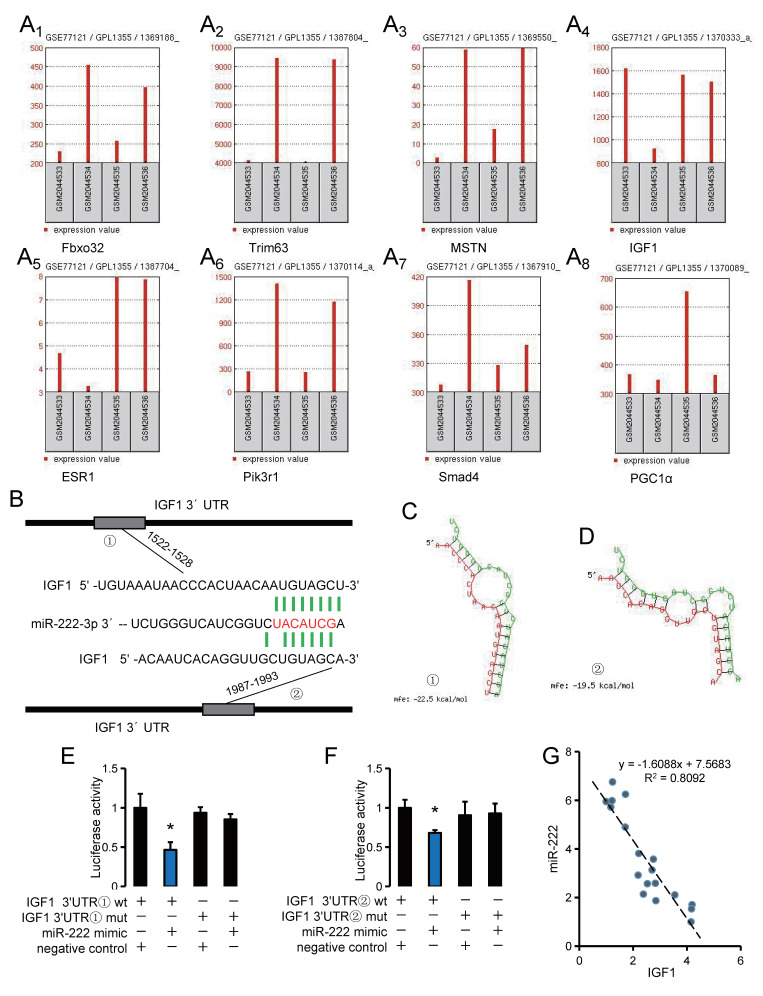
IGF1 is a target gene of miR-222. (**A_1_**–**A_8_**) Expression signature of muscle atrophy marker genes and node genes (miR-222 target gene interaction network) in a denervated muscle atrophy model. Data were sourced from the GEO database (GSE77121, GSM2044533: Control diet_Sham tretment, GSM2044534: Control diet_Denervated treatment, GSM2044535: Genistein diet_Sham treatment, GSM2044536: Genistein diet_Denervated treatment) and analyzed online using the GEOR2 tool. (**B**–**D**) Prediction and secondary structure analysis of *IGF1* and miR-222 binding sites. (**E**,**F**) Fluorescence intensity of a dual-luciferase reporter assay system for two potential binding sites of miR-222 and *IGF1*. (**G**) Correlation analysis of miR-222 and *IGF1* expression levels in TA muscle. C, F_G, *n* = 3; H, *n* = 18. * *p* < 0.05, compared with the NC group.

miRNAs mainly exert biological functions by regulating target genes. We further analyzed and verified the possible target genes of miR-222 in regulating muscle atrophy. The interaction network analysis of miR-222 target genes found that there were four genes related to muscle development and at the nodes of the interaction network: *Tcf712*, *Psma8*, *Gnai2*, *ESR1*, *Smad4*, *Pik3r1* and *IGF1* (Figure 4). Using data from public databases, we analyzed the expression patterns of muscle atrophy marker genes and network node molecules in a denervated muscular atrophy animal model of dietary genistein. The results showed that muscle tissue denervation resulted in a significant up-regulation of *Fbxo32*, *Trim63*, *MSTN*, *Pik3r1* and *smad4*, and a significant decrease in *IGF1* and *ESR1*. Dietary genistein alleviated denervation-induced upregulation of *Fbxo32*, *pik3r1*, and *smad4*, while reversing the downregulation of *IGF1* and *ESR1* (Figure 5A). miRNAs mainly inhibit the post-transcriptional expression of target genes, while miR-222 is up-regulated in muscle atrophy, so we speculate that miR-222 may regulate muscle atrophy through *IGF1* and *ESR1*, which are down-regulated in muscle atrophy. Further analysis showed that *IGF1* has a higher expression level in skeletal muscle than *ESR1*, and there are two potential binding sites for miR-222 in the 3’UTR of *IGF1*, suggesting that *IGF1* may play a more important role in miR-222-regulated skeletal muscle atrophy (Figure 5B–D). The dual-luciferase reporter system showed that both binding sites of the 3’UTR of *IGF1* could bind to miR-222 (Figure 5E,F). Furthermore, correlation analysis showed that the expressionof miR-222 and *IGF1* were highly negatively correlated (Figure 5G).

### 3.5. Genistein Attenuates Dexamethasone-Induced Muscle Atrophy by Downregulating miR-222 In Vivo and In Vitro

**Figure 6:**
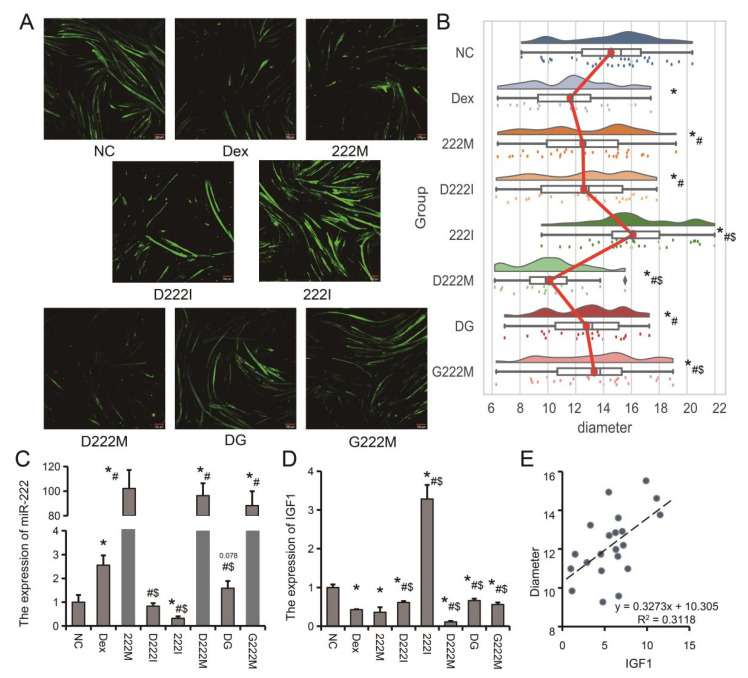
Effects of combined treatment of genistein and miR-222 on in vitro muscle atrophy model. (**A**) Immunofluorescence staining of *MyHC*. Negative control (NC), dexamethasone (Dex), miR-222 mimic (222M), miR-222 inhibitor (222I), dexamethasone + miR-222 mimic (D222M), dexamethasone + miR-222 inhibitor (D222I), dexamethasone + genistein (DG), genistein + miR-222 mimic (G222M). (**B**) Rain-cloud diagram showing C2C12 myotube diameter distribution. (**C**) The expression of miR-222 in C2C12 myotubes. (**D**) The expression of *IGF1* in C2C12 myotubes. (**E**) Correlation analysis of miR-222 and *IGF1* expression levels in C2C12 myotubes. *n* = 3. * *p* < 0.05, compared with the NC group, # *p* < 0.05, compared with Dex group, $ *p* < 0.05, compared with 222M group.

To further analyze the role of miR-222 during muscle atrophy and the regulation of miR-222 expression by genistein, co-treatment of C2C12 myoblasts with genistein, miR-222 mimic (222M), miR-222 (222I) inhibitor were performed. The results showed: (1) In the single treatment group, compared with NC, Dex and miR-222 mimic significantly reduced the diameter of myotubes, while miR-222 inhibitor significantly increased the diameter of myotubes; (2) In the muscle atrophy model, compared with the Dex group, both genistein and miR-222 inhibitor could alleviate the decrease in the diameter of myotubes caused by Desemide treatment, while miR-222 mimic further aggravated the decrease in the diameter of myotubes; (3) Compared with miR-222 mimic alone, genistein could alleviate the reduction of myotube diameter caused by miR-222 overexpression (Figure 6A,B). The expression of *IGF1* in C2C12 myotubes decreased with the increase of miR-222 expression level (Figure 6C,D). Compared with the Dex group, the DG group significantly down-regulated the expression of miR-222 (Figure 6C). We also found that *IGF1* expression level was positively correlated with myotube diameter (Figure 6E). These results suggest that promoting miR-222 expression in C2C12 myotubes exacerbates muscle atrophy, while reducing miR-222 expression can effectively alleviate muscle atrophy.

**Figure 7:**
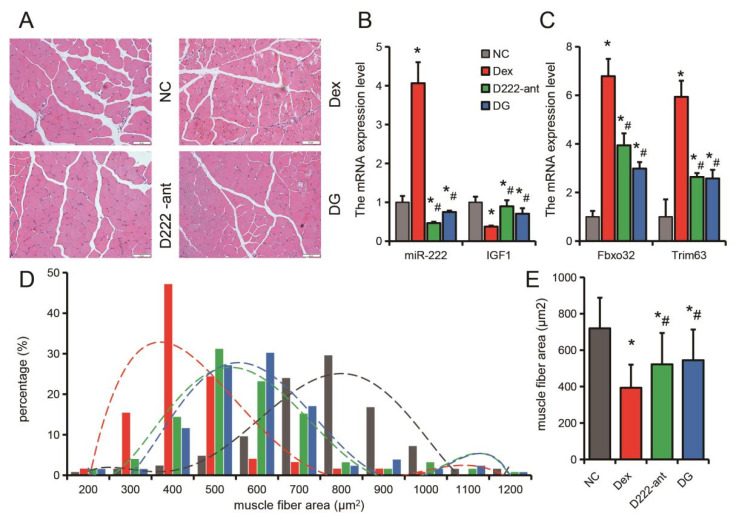
Genistein and miR-222 antagomiR alleviate Dex-induced muscle atrophy in vivo. (**A**) HE-stained section of TA muscle. (**B**) The expression of miR-222 and *IGF1* in TA muscle. (**C**) The expression of *Fbxo32* and *Trim63* in TA muscle. (**D**) Distribution frequency of TA muscle fiber area. (**E**) Mean muscle fiber area of TA muscle. NC: negative control; Dex: dexamethasone; D222-ant: dexamethasone + antagomiR-222; DG: dexamethasone + genistein. *n* = 4. * *p* < 0.05, compared with the NC group, # *p* < 0.05.

In a Dex-induced muscle atrophy model in mice, injection of miR-222 antagomiR or genistein treatment had the same effect. Injection of miR-222 antagomiR or treatment with genistein could effectively inhibit the up-regulation of miR-222, *Fbxo32* and *Trim63* induced by Dex, and slow down the down-regulation of *IGF1* induced by Dex (Figure 7A–C). Furthermore, injection of miR-222 antagomiR or treatment with genistein significantly inhibited the decrease in muscle fiber area caused by Dex (Figure 7D,E). These results suggest that both genistein and miR-222 can regulate muscle atrophy. miR-222 plays an important role for genistein in alleviating muscle atrophy.

## 4. Discussion

Muscle atrophy is often caused by a variety of stressors, is debilitating and severely affects quality of life [27]. Studies have found that miRNAs are abnormally expressed in skeletal muscle atrophy, but the specific mechanisms of these miRNAs remain unclear [17]. Here, we report that miR-222 is upregulated in multiple types of muscle atrophy, and genistein acts as a natural regulator of miR-222 to alleviate muscle atrophy.

The stress response caused by various factors is an important influencing factor of skeletal muscle atrophy. Glucocorticoids (GCs) are the most important regulators of the body’s stress response [28]. It is also the most widely used and effective anti-inflammatory and immunosuppressant in clinical practice [29]. GCs are often the first choice in emergency or critical situations. Dexamethasone (Dex) is a synthetic long-acting glucocorticoid that has been widely used and reported on the new coronavirus pneumonia [30]. However, excess glucocorticoids can significantly reduce muscle strength and motor activity, leading to decreased muscle mass and muscle atrophy [13,31]. In this study, it was found that the body weight of the mice decreased significantly after 3 days of Dex injection, and the weight of the TA muscle was also significantly decreased after 7 days. Interestingly, we found that Dex had a stronger effect on TA muscle than Sol muscle. TA muscle fiber area decreased by 42.34% and Sol muscle decreased by 35.75% after Dex treatment. In cellular models, we also found that *MYH4*, a marker for rapidly contracting myofibers [32], had the greatest decrease in both expression and proportion. Therefore, our follow-up study mainly focused on the tibialis anterior (TA) muscle. Previous studies have also found that glucocorticoids mainly affect fast-twitch muscle fibers [33].

Estrogen or hormone replacement therapy has been shown to have beneficial effects on muscle mass and muscle wasting [12]. Estrogen mainly exerts its biological effects through two receptors, ESR1 and ESR2 [34]. ESR1 and ESR2 are ubiquitous in female and male muscle and expressed in muscle fibers, endothelial cells, and satellite cells [35,36]. Studies have found that deletion of ESR1 in skeletal muscle leads to impaired skeletal muscle glucose metabolism [37] and reduced muscle oxidative metabolism [38]. In addition, ESR1 expression was down-regulated in atrophic skeletal muscle [16,39], and endurance training increased ESR1 expression in skeletal muscle [36,40]. Genistein is a phytoestrogen with estrogen-like effects in animals [41]. In this study, it was found that genistein could inhibit the up-regulation of *MSTN*, *Fbxo32* and *Trim63* and the down-regulation of *IGF1* induced by Dex, and relieve muscle atrophy in vitro and in vivo. Upregulation of *MSTN*, *Fbxo32*, and *Trim63*, and downregulation of *IGF1* have been reported to be common features in starvation, disuse, denervation, and glucocorticoid-induced muscle atrophy, so these genes are also frequently used as markers of muscle atrophy [7,22]. Our results suggest that the improvement of muscle atrophy by genistein may be generalized in multiple models.

The specific miRNAs expressed in muscle are called myomiRs [42]. myomiRs have been widely reported to be involved in skeletal muscle development and maintenance of physiological function [43,44]. Most of the previously reported miRNAs involved in muscle atrophy also belong to myomiRs [45]. With the widespread application of high-throughput sequencing technology, more and more non-myomiRs have been found to be related to muscle atrophy, such as miR-29b [22], miR-497-5p [46], miR-27 [47], etc. In this study, we performed a combined analysis of differentially expressed miRNAs in models of aging, disuse, and denervation-induced muscle atrophy. After co-expression analysis, miR-540-3p, miR-221-5p and miR-181b-1-3p were found to be down-regulated in 3 muscle atrophy models, while miR-222-3p, miR-342-3p and miR-92a- 1-5p were upregulated in 3 models of muscle atrophy. Among them, miR-222-3p was found to be regulated by genistein in our previous study [19]. In this study, it was also found that genistein could down-regulate the Dex-induced increase in miR-222 expression both in vitro and in vivo. Through GO enrichment analysis of miR-222 target genes, and found that miR-222 target genes are involved in many biological processes related to skeletal muscle development, suggesting that miR-222 may be the core miRNA that regulates muscle atrophy.

In order to further clarify the target genes of miR-222 that play a major role in muscle atrophy, we analyzed the interaction regulatory network of miR-222 target genes, and the results showed that *Tcf712*, *Psma8*, *Gnai2*, *ESR1*, *Smad4*, *Pik3r1* and *IGF1* are important molecular nodes, among which *ESR1* [16], *IGF1* [7] and *Pik3r1* [48] are closely related to skeletal muscle atrophy. Usually, miRNAs exert biological functions by inhibiting the expression of target genes, here we found that miR-222 is up-regulated in muscle atrophy, and *Pik3r1* is also highly expressed after muscle atrophy, so *Pik3r1* was not further studied. Furthermore, it is worth noting that we found that *PGC1α*, identified in our previous study as a target gene of miR-222 to regulate muscle fiber turnover, was unchanged in models of muscle atrophy [19]. It suggested that *PGC1α* may not play a major role in muscle atrophy involving mir-222. This phenomenon, which is differentially characterized in different models, has also been found in other studies [22].

Through sequence alignment analysis, we found that there are two potential binding sites for miR-222 in the 3’UTR of *IGF1*, which were subsequently verified using a dual-luciferase reporter system. *IGF1* is one of the core molecules regulating skeletal muscle atrophy, mainly by promoting muscle protein synthesis and inhibiting muscle atrophy [49,50]. In addition, miR-222 has a high negative correlation with the expression of *IGF1*, and unlike miR-222 expression, genistein can inhibit the down-regulation of *IGF1* caused by Dex. Similar to the in vitro results, injection of miR-222 antagomiR and genistein treatment both effectively alleviated Dex-induced muscle atrophy. These results suggest that genistein alleviates muscle atrophy by promoting *IGF1* expression by downregulating miR-222.

## 5. Conclusions

Taken together, our results suggest that genistein may act as a natural regulator of miR-222 for the prevention and treatment of dexamethasone-induced muscle atrophy. miR-222 is upregulated in multiple types of muscle atrophy and regulates muscle atrophy progression by targeting *IGF1*. However, genistein could inhibit dexamethasone or miR-222 overexpression-induced muscle atrophy. Genistein may be used in the future to treat muscle atrophy or related diseases caused by dysregulation of miR-222.

## Data Availability

For the remaining data that may be relevant, the corresponding authors can be contacted.

## Supplementary Materials

The following supporting information can be downloaded at: https://www.mdpi.com/article/10.3390/nu14091861/s1, Table S1: Sequences of PCR primers used in this study; Table S2: GO analysis results of miR-222 target genes.

## References

[1] Shen L., Du J., Xia Y., Tan Z., Fu Y., Yang Q., Li X., Tang G., Jiang Y., Wang J. (2016). Genome-wide landscape of DNA methylomes and their relationship with mrna and mirna transcriptomes in oxidative and glycolytic skeletal muscles. Sci. Rep..

[2] Pedersen B.K., Febbraio M.A. (2012). Muscles, exercise and obesity: Skeletal muscle as a secretory organ. Nat. Rev. Endocrinol..

[3] Zhang H., Liang J., Chen N. (2020). Do not neglect the role of circadian rhythm in muscle atrophy. Ageing Res. Rev..

[4] Siff T., Parajuli P., Razzaque M., Atfi A. (2021). Cancer-mediated muscle cachexia: Etiology and clinical management. Trends Endocrinol. Metab. TEM.

[5] Yin L., Li N., Jia W., Wang N., Liang M., Yang X., Du G. (2021). Skeletal muscle atrophy: From mechanisms to treatments. Pharmacol. Res..

[6] Jang Y., Rodriguez K., Lustgarten M., Muller F., Bhattacharya A., Pierce A., Choi J., Lee N., Chaudhuri A., Richardson A. (2020). Superoxide-mediated oxidative stress accelerates skeletal muscle atrophy by synchronous activation of proteolytic systems. GeroScience.

[7] Stitt T., Drujan D., Clarke B., Panaro F., Timofeyva Y., Kline W., Gonzalez M., Yancopoulos G., Glass D. (2004). The igf-1/pi3k/akt pathway prevents expression of muscle atrophy-induced ubiquitin ligases by inhibiting foxo transcription factors. Mol. Cell.

[8] Mc Pherron A., Lawler A., Lee S. (1997). Regulation of skeletal muscle mass in mice by a new tgf-beta superfamily member. Nature.

[9] Cid-Díaz T., Santos-Zas I., González-Sánchez J., Gurriarán-Rodríguez U., Mosteiro C., Casabiell X., García-Caballero T., Mouly V., Pazos Y., Camiña J. (2017). Obestatin controls the ubiquitin-proteasome and autophagy-lysosome systems in glucocorticoid-induced muscle cell atrophy. J. Cachexia Sarcopenia Muscle.

[10] Zhao J., Zhai B., Gygi S., Goldberg A. (2015). Mtor inhibition activates overall protein degradation by the ubiquitin proteasome system as well as by autophagy. Proc. Natl. Acad. Sci. USA.

[11] Sukari A., Muqbil I., Mohammad R., Philip P., Azmi A. (2016). F-box proteins in cancer cachexia and muscle wasting: Emerging regulators and therapeutic opportunities. Semin. Cancer Biol..

[12] Martín A., Priego T., López-Calderón A. (2018). Hormones and muscle atrophy. Adv. Exp. Med. Biol..

[13] Schakman O., Kalista S., Barbé C., Loumaye A., Thissen J. (2013). Glucocorticoid-induced skeletal muscle atrophy. Int. J. Biochem. Cell Biol..

[14] Su Z., Hu L., Cheng J., Klein J., Hassounah F., Cai H., Li M., Wang H., Wang X. (2016). Acupuncture plus low-frequency electrical stimulation (acu-lfes) attenuates denervation-induced muscle atrophy. J. Appl. Physiol..

[15] Zhao H., Zhou L., Li L., Coon V.J., Chatterton R., Brooks D., Jiang E., Liu L., Xu X., Dong Z. (2018). Shift from androgen to estrogen action causes abdominal muscle fibrosis, atrophy, and inguinal hernia in a transgenic male mouse model. Proc. Natl. Acad. Sci. USA.

[16] Aoyama S., Jia H., Nakazawa K., Yamamura J., Saito K., Kato H. (2016). Dietary genistein prevents denervation-induced muscle atrophy in male rodents via effects on estrogen receptor-α. J. Nutr..

[17] Liu Q., Deng J., Qiu Y., Gao J., Li J., Guan L., Lee H., Zhou Q., Xiao J. (2021). Non-coding rna basis of muscle atrophy. Mol. Ther. Nucleic Acids.

[18] Majid S., Dar A., Saini S., Chen Y., Shahryari V., Liu J., Zaman M., Hirata H., Yamamura S., Ueno K. (2010). Regulation of minichromosome maintenance gene family by microrna-1296 and genistein in prostate cancer. Cancer Res..

[19] Gan M., Shen L., Liu L., Guo Z., Wang S., Chen L., Zheng T., Fan Y., Tan Y., Jiang D. (2020). Mir-222 is involved in the regulation of genistein on skeletal muscle fiber type. J. Nutr. Biochem..

[20] Gan M., Shen L., Fan Y., Tan Y., Zheng T., Tang G., Niu L., Zhao Y., Chen L., Jiang D. (2019). Microrna-451 and genistein ameliorate nonalcoholic steatohepatitis in mice. Int. J. Mol. Sci..

[21] Gan M., Shen L., Wang S., Guo Z., Zheng T., Tan Y., Fan Y., Liu L., Chen L., Jiang A. (2020). Genistein inhibits high fat diet-induced obesity through mir-222 by targeting btg2 and adipor1. Food Funct..

[22] Jin L., Chan M.C., Yan Y., Bei Y., Ping C., Zhou Q., Cheng L., Lei C., Ziegler O., Rowe G.C. (2017). Mir-29b contributes to multiple types of muscle atrophy. Nat. Commun..

[23] Son Y.H., Jang E.J., Kim Y.W., Lee J.H. (2017). Sulforaphane prevents dexamethasone-induced muscle atrophy via regulation of the akt/foxo1 axis in c2c12 myotubes. Biomed. Pharmacother..

[24] Agarwal V., Bell G., Nam J., Bartel D. (2015). Predicting effective microrna target sites in mammalian mrnas. Elife.

[25] Krüger J., Rehmsmeier M. (2006). Rnahybrid: Microrna target prediction easy, fast and flexible. Nucleic Acids Res..

[26] Chen Y., Wang X. (2020). Mirdb: An online database for prediction of functional microrna targets. Nucleic Acids Res..

[27] Gheller B., Riddle E., Lem M., Thalacker-Mercer A. (2016). Understanding age-related changes in skeletal muscle metabolism: Differences between females and males. Annu. Rev. Nutr..

[28] Praestholm S.M., Correia C.M., Grntved L. (2020). Multifaceted control of gr signaling and its impact on hepatic transcriptional networks and metabolism. Front. Endocrinol..

[29] Vandewalle J., Luypaert A., De Bosscher K., Libert C. (2018). Therapeutic mechanisms of glucocorticoids. Trends Endocrinol. Metab. TEM.

[30] Mehta H., An H., Andersen K., Mansour O., Madhira V., Rashidi E., Bates B., Setoguchi S., Joseph C., Kocis P. (2021). Use of hydroxychloroquine, remdesivir, and dexamethasone among adults hospitalized with covid-19 in the united states: A retrospective cohort study. Ann. Intern. Med..

[31] Yoshioka Y., Samukawa Y., Yamashita Y., Ashida H. (2020). 4-hydroxyderricin and xanthoangelol isolated from angelica keiskei prevent dexamethasone-induced muscle loss. Food Funct..

[32] Fitts R., Widrick J. (1996). Muscle mechanics: Adaptations with exercise-training. Exerc. Sport Sci. Rev..

[33] Seene T., Viru A. (1982). The catabolic effect of glucocorticoids on different types of skeletal muscle fibres and its dependence upon muscle activity and interaction with anabolic steroids. J. Steroid Biochem. Mol. Biol..

[34] Dayton W., White M. (2014). Meat science and muscle biology symposium-role of satellite cells in anabolic steroid-induced muscle growth in feedlot steers. J. Anim. Sci..

[35] Wiik A., Ekman M., Johansson O., Jansson E., Rnsson M. (2009). Expression of both oestrogen receptor alpha and beta in human skeletal muscle tissue. Histochem. Cell Biol..

[36] Wiik A.T., Gustafsson T., Esbjörnsson M., Johansson O., Jansson E. (2010). Expression of oestrogen receptor alpha and beta is higher in skeletal muscle of highly endurance-trained than of moderately active men. Acta Physiol..

[37] Barros R., Machado U.F., Warner M., Gustafsson J.A. (2006). Muscle glut4 regulation by estrogen receptors erβ and erα. Proc. Natl. Acad. Sci. USA.

[38] Hevener A., Ribas V., Moore T., Zhou Z. (2020). The impact of skeletal muscle erα on mitochondrial function and metabolic health. Endocrinology.

[39] Park K.-S., Kim H., Kim H.J., Lee K.-I., Lee S.-Y., Kim J. (2022). Paeoniflorin alleviates skeletal muscle atrophy in ovariectomized mice through the erα/nrf1 mitochondrial biogenesis pathway. Pharmaceuticals.

[40] Soleymani T., Daraei P., Ribas V., Drew B., Hevener A. (2009). Erα Is Elevated with Endurance Exercise and Is Critical for Normal Skeletal Muscle Oxidative Metabolism and Insulin Action.

[41] Yin X.U., Guan J. (2010). Research progress on biological function of genistein. Med. Recapitul..

[42] Zilahi E., Adamecz Z., Bodoki L., Griger Z., Póliska S., Nagy-Vincze M., Dankó K. (2019). Dysregulated expression profile of myomirs in the skeletal muscle of patients with polymyositis. Ejifcc.

[43] Srivastava S., Rathor R., Singh S., Suryakumar G. (2021). Emerging role of myomirs as biomarkers and therapeutic targets in skeletal muscle diseases. Am. J. Physiol. Cell Physiol..

[44] McCarthy J. (2011). The myomir network in skeletal muscle plasticity. Exerc. Sport Sci. Rev..

[45] Marinho R., Alcântara P., Ottoch J., Seelaender M. (2017). Role of exosomal micrornas and myomirs in the development of cancer cachexia-associated muscle wasting. Front. Nutr..

[46] Freire P., Cury S., Lopes L., Fernandez G., Liu J., de Moraes L., de Oliveira G., Oliveira J., de Moraes D., Cabral-Marques O. (2021). Decreased mir-497-5p suppresses il-6 induced atrophy in muscle cells. Cells.

[47] Yang X., Li Z., Wang Z., Yu J., Ma M., Nie Q. (2022). Mir-27b-3p attenuates muscle atrophy by targeting cbl-b in skeletal muscles. Biomolecules.

[48] Chen T., Kuo T., Dandan M., Lee R., Chang M., Villivalam S., Liao S., Costello D., Shankaran M., Mohammed H. (2021). The role of striated muscle pik3r1 in glucose and protein metabolism following chronic glucocorticoid exposure. J. Biol. Chem..

[49] Schiaffino S., Dyar K., Ciciliot S., Blaauw B., Sandri M. (2013). Mechanisms regulating skeletal muscle growth and atrophy. FEBS J..

[50] Brooks N., Myburgh K. (2014). Skeletal muscle wasting with disuse atrophy is multi-dimensional: The response and interaction of myonuclei, satellite cells and signaling pathways. Front. Physiol..

